# Exercise Alleviates Aging of Adipose Tissue through Adipokine Regulation

**DOI:** 10.3390/metabo14030135

**Published:** 2024-02-22

**Authors:** Dandan Jia, Huijie Zhang, Tiemin Liu, Ru Wang

**Affiliations:** 1 School of Exercise and Health, Shanghai Frontiers Science Research Base of Exercise and Metabolic Health, Shanghai University of Sport, Shanghai 200438, China; 2321518039@sus.edu.cn; 2 State Key Laboratory of Genetic Engineering, School of Life Sciences, Fudan University, Shanghai 200438, China; tiemin_liu@fudan.edu.cn

**Keywords:** exercise, adipose tissue, aging, adipokines

## Abstract

Adipose tissue undergoes changes with aging, leading to increased adiposity, inflammatory cell infiltration, reduced angiogenesis, heightened oxidative stress, and alterations in its metabolic function. Regular exercise has been recognized as a powerful intervention that can positively influence adipose tissue health and mitigate the effects of aging. However, the molecular mechanisms underlying the benefits of regular exercise on aging adipose tissue function remain poorly understood. Adipokines released through regular exercise play a potential role in mitigating adipose tissue aging, enhancing the metabolism of glucose and lipids, reducing inflammation and fibrosis, and promoting fat browning and thermogenesis. This review comprehensively summarizes the benefits of regular exercise in addressing the age-related decline in adipose tissue function. Utilizing relevant examples of this approach, we address the possibility of designing therapeutic interventions based on these molecular mechanisms.

## 1. Introduction

The prevalence of obesity, coupled with its profound influence on the demographic composition of the global population, has experienced a notable and concerning escalation over the last four decades. The most recent national prevalence figures for the years 2015–2019, following Chinese criteria, indicate rates of 3.6% for obesity in children under 6 years, 7.9% for obesity in children and adolescents aged 6–17 years, and 16.4% for obesity in adults (≥18 years) [1]. The study revealed a connection between obesity and the aging process. However, the prevalence of obesity in older people has dramatically increased in recent years, with more than 30% of individuals aged 60 and over being overweight or obese [2]. Further research is imperative to comprehend the morphological and molecular alterations associated with age in adipose tissue (AT), aiming to address and combat age-related metabolic diseases.

Aging adipose depots exhibit heightened infiltration of inflammatory cells, enlarged lipid droplets, and an increased prevalence of senescent cells [3]. These age-related changes in AT result in a reduced basal metabolic rate, impaired insulin responsiveness, elevated ectopic deposition of lipids, and consequent lipotoxicity. Emerging evidence suggests that exercise (e.g., resistance exercise, endurance exercise) is a highly effective intervention in alleviating obesity and plays a significant role in individual metabolism, as evidenced by its impact on the morphology and function of adipose depots [4,5,6,7,8,9]. Moreover, circulating factors induced by exercise (e.g., swimming exercise, voluntary wheel-running exercise), known as exerkines, are involved in the metabolism of AT in response to aging [7,10,11,12,13]. The goal of this review is to offer a comprehensive overview of the benefits of regular exercise in counteracting age-related declines in AT function. This includes addressing issues such as adipose expansion, decreased vascularity and mitochondrial function, fibrosis, and inflammatory cell infiltration. The relevance of regular exercise in mitigating metabolic disorders associated with aging AT will also be discussed.

## 2. Morphological Changes in Aged Adipose Tissue

**Figure 1:**
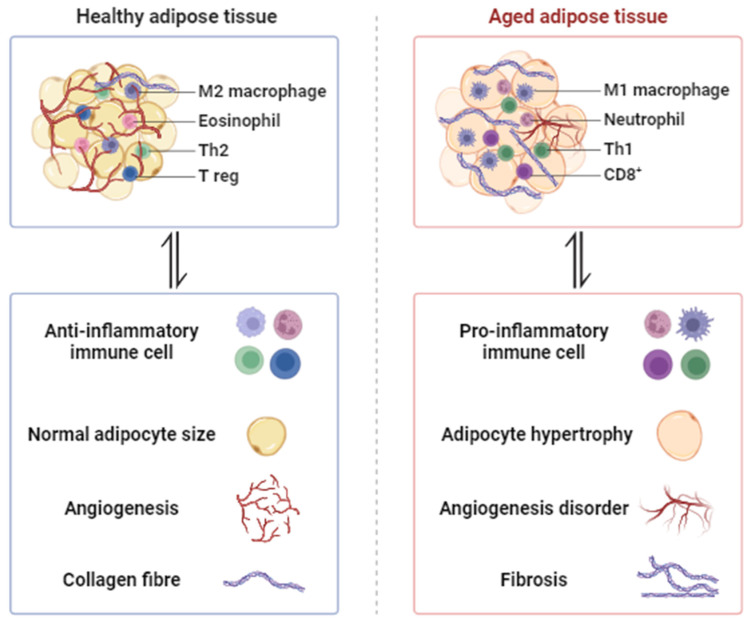
The morphological changes in aging adipose tissue. Adipose plasticity becomes compromised with age, leading to adipocyte hypertrophy, a decline in adipogenesis, decreased angiogenesis, increased fibrosis, pro-inflammatory macrophage infiltration (M1 macrophage, Neutrophil, Th1, CD8^+^), and decreased anti-inflammatory macrophage infiltration (M2 macrophage, Eosinophil, Th2, T reg).

AT, an extraordinarily flexible and heterogeneous organ, plays a crucial role in regulating immune responses, body temperature, energy balance, insulin sensitivity, and overall physiological functions [14]. AT exhibits an extraordinary capacity to adapt to a range of internal and external signals, owing to its high degree of plasticity [14]. Nevertheless, a newfound understanding of the cellular and functional remodeling of white adipose tissue (WAT) and brown adipose tissue (BAT) during aging has surfaced in recent years. Adipose plasticity becomes compromised with age, as indicated by heightened visceral adiposity, reduced lipolysis and thermogenesis, and an inability to maintain body temperature during cold stress [15,16]. Current endeavors focus on investigating the potential underlying mechanisms behind age-related alterations in AT, including hypertrophy, adipogenesis, hypoxia, angiogenesis, fibrosis, inflammation, mitochondrial biogenesis, and function [17] (Figure 1).

### 2.1. Hypertrophy and Adipogenesis Declines

AT exhibits a significant degree of plasticity and plays a role in influencing metabolism during both health and aging in response to various physiological stimuli. These stimuli include obesity, diabetes, fasting, fatty liver, cardiometabolic disease, cold exposure, local hyperthermia, and prolonged exercise [14,17]. With advancing age, the plasticity of adipose tissue becomes compromised [18], affecting the ability of preadipocytes to self-renew and the replication of adipocyte progenitors in the stromovascular fraction (SVF) [19]. Adipocytes undergo expansion as body weight increases with age. Hypertrophic adipocytes exhibit reduced expression of fat identity genes, compromising their ability to store excess lipid and releasing inflammatory adipokines that exacerbate the adipose tissue microenvironment [20]. Excessive enlargement of WAT and inadequate angiogenesis result in cellular hypoxia, triggering a pro-inflammatory response. This cascade effect diminishes adipogenesis, promotes fibrosis, and hampers metabolic flexibility and thermogenesis in aging and age-related diseases [21,22,23]. BAT, characterized by multilocular fat droplets and abundant mitochondria, serves as a thermogenic energy-expending tissue. It regulates body temperature through the mediation of mitochondrial uncoupling protein 1 (UCP1) in response to aging and age-related diseases [24,25]. The activation of brown or beige adipocytes contributes to alleviating metabolic disorders [26]. 

With advancing age, the decline in adipogenic potential can be associated with cellular senescence, as indicated by elevated markers of senescence in WAT depots, such as p16^Ink4a^ and senescence-associated beta-galactosidase activity [27]. The activation of the senescent pathway may compromise adipogenesis. Adipose-derived stem cells from older donors exhibited heightened expression of p16^Ink4a^, which significantly contributes to reduced cellular differentiation [28]. However, cellular senescence, among other aging-related processes, influences the endocrine function of AT. Functional WAT releases various factors that contribute to maintaining energy homeostasis, such as leptin, resistin, chemerin, and adiponectin. Furthermore, the secretion of these adipokines is affected by the aging process. 

### 2.2. Hypoxia and Angiogenesis Disorder

The excessive enlargement of WAT and inadequate angiogenesis create a hypoxic environment in cells in response to obesity. This condition leads to a pro-inflammatory response and disorder in angiogenesis. With aging and obesity, the reduced availability of oxygen can trigger cellular hypoxia and inflammation, contributing to local and systemic metabolic dysfunction. Hypoxia-inducible factors (HIFs) play a role in various cellular functions, including glucose utilization, angiogenesis, apoptosis, extracellular matrix (ECM) remodeling, recruitment of macrophages, and fibrosis [29,30]. The hypertrophic growth associated with aging results in reduced oxygen diffusion, exacerbated by insufficient compensation from the vasculature. Despite the absence of angiogenesis, HIF-1α seems to be upregulated in aged AT. However, the instability of the HIF-1α protein can pose a challenge to quantification [31,32]. Furthermore, HIF-1α plays a role in mitochondrial biogenesis and function in aged AT. Mitochondrial complex IV (CIV) activity and assembly are already suppressed in white adipocytes of middle-aged mice, involving a HIF1α-dependent decline of essential CIV components, such as COX5B [31]. 

### 2.3. Fibrosis

Fibrosis has been recognized as a hallmark of dysfunctional AT in aging and obesity. It is a common pathological consequence of ECM dysregulation and arises from an imbalance between the synthesis and degradation of ECM fibrillar components [32]. However, the excessive deposition of collagen in AT triggers persistent and chronic inflammation, ultimately disrupting AT homeostasis and exacerbating metabolic dysfunction in aging and obesity [33,34]. Importantly, AT fibrosis is linked to insulin resistance in individuals with obesity [35,36]. The regulation of AT fibrosis involves hypoxia, which induces the transcription of ECM components and alters cellular redox status to impact collagen crosslinking enzymes such as lysyl oxidase [36]. Furthermore, unresolved inflammation is frequently linked to the progression of fibrosis in various pathological conditions [37]. Mechanistically, the activation of macrophage toll-like receptor 4 (TLR4) recruits macrophage-inducible C-type lectin, stimulating pathways involved in ECM production and degradation, as well as fibroblast proliferation and differentiation [38]. Additionally, the accumulation of fibrosis in subcutaneous WAT is associated with resistance to weight loss one year after bariatric surgery [39]. BAT can selectively release various cytokines to counteract fibrosis when transplanted into WAT, achieved by upregulating lipogenesis and fatty acid metabolism [40].

### 2.4. Inflammation

Adipose tissue exhibits an enrichment of proinflammatory macrophages in response to both obesity and aging [41]. During the aging process, visceral adiposity is frequently linked to changes in AT leukocytes, inflammation, and metabolic dysfunction. In contrast to obesity, the accumulation of inflammatory factors with age is not dependent on macrophage abundance, as evidenced by the lack of increase in the number of macrophages with age. Indeed, aging regulates macrophage polarization by activating TLR4 signaling and influencing transcript levels of inflammatory IL-6 and monocyte chemoattractant protein 1 (MCP-1). Aging is additionally linked to an expansion of resident immune cells in AT, including B and T cells, which exhibit distinct transcriptional profiles compared to age-related splenic B and T cells [42,43]. Studies have demonstrated that mice lacking fat-resident regulatory T cells are safeguarded against age-related insulin resistance, although they remain vulnerable to insulin resistance and metabolic diseases associated with obesity [43]. Furthermore, inhibiting NLRP3-dependent B cell accumulation can reverse metabolic impairment in aged AT [42]. 

## 3. Therapeutic Approaches to Enhance Aging Adipose Tissue

### 3.1. Cold Exposure

**Figure 2:**
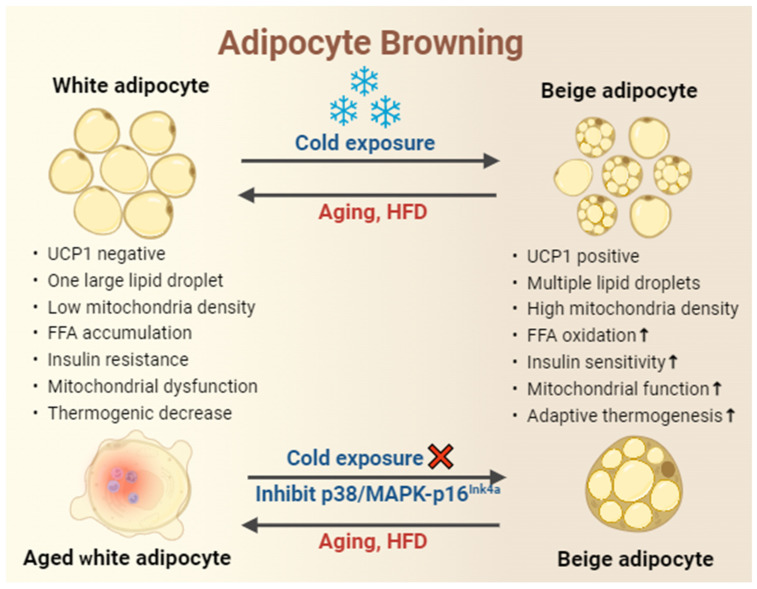
Cold exposure enhances adipocyte browning in response to obesity but not in age-related obesity. Cold exposure is implicated in the prevention and management of obesity, as evidenced by the increased expression of UCP1, reduced accumulation of large lipid droplets, enhanced mitochondrial biogenesis and function, promotion of FFA oxidation, increased insulin sensitivity, and improvement of white adipocyte browning and thermogenesis in WAT. Nevertheless, the potential to form cold-induced beige adipocytes declines with age. In contrast, reversing cellular aging through the p38/MAPK-p16^Ink4a^ pathway rejuvenates cold-induced beiging. HFD, high-fat diet.

Environmental cold exposure triggers the formation of mitochondria-rich and thermogenic beige adipocytes in WAT, a process known as browning [44,45] (Figure 2). It has been reported that cold exposure is a remarkably potent stimulus for enhancing insulin sensitivity and glucose and lipid metabolism. This occurs through the reduction of large lipid droplet accumulation, clearance of serum triacylglycerol, promotion of FFA oxidation, and the delivery of long-chain fatty acids. These actions contribute to increased expression of UCP1, improvement of mitochondrial biogenesis and function, and enhancement of browning in white adipocytes within WAT [44,46,47]. BAT is characterized by its capacity to dissipate energy as heat through the action of UCP1, which is activated by the sympathetic nervous system (SNS) during activities such as exercise or exposure to cold [8,48,49]. Nevertheless, triggering the senescence pathway in young beige progenitors induces premature cellular senescence and hinders their potential to form cold-induced beige adipocytes. On the contrary, genetically or pharmacologically reversing cellular aging through the p38/MAPK-p16^Ink4a^ pathway in aged mouse or human beige progenitor cells rejuvenates cold-induced beiging [50].

### 3.2. Local Hyperthermia Therapy

**Figure 3:**
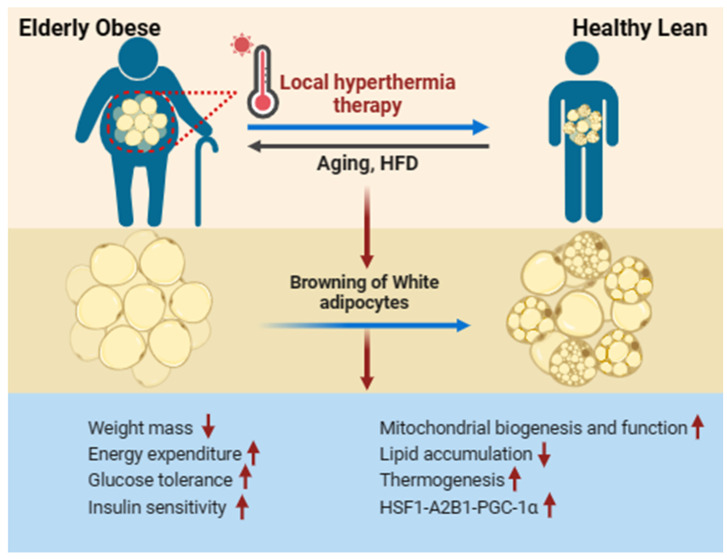
Local hyperthermia therapy improves WAT browning in aging-induced obese. Local hyperthermia therapy stimulates the activation and production of beige adipocytes in individuals over the age of 65 with obesity, enhancing metabolic performance. This includes reductions in lipid accumulation and body mass, improvements in diabetic neuropathic symptoms, enhanced glucose tolerance, increased insulin sensitivity, and the promotion of mitochondrial biogenesis and thermogenesis. These effects are achieved through the activation of the HSF1-A2BA-PGC-1α pathway. Red arrows indicate an increase (up arrow) or a decrease (down arrow).

Earlier research has demonstrated that cold exposure or activation of adrenergic signaling can be a beneficial method for promoting the generation of beige adipose tissue [26,51]. Conversely, these treatments have limited applications due to associated cardiovascular risks [52,53,54,55]. Recent studies have highlighted that local hyperthermia therapy could offer promising scientific benefits and serve as a potential therapeutic approach for aging-related diseases [56,57]. The underlying molecular mechanism behind these positive outcomes of hyperthermia therapy involves the expression of heat shock protein 72 (HSP72), a classic stress-responsive protein that plays a role in stabilizing intracellular proteins. This mechanism is supported by evidence demonstrating enhanced glucose tolerance and insulin resistance, improved mitochondrial function, and a reduction in lipid accumulation [58]. Recent studies have suggested that local hyperthermia therapy stimulates thermogenesis, enhances fat metabolism, and boosts the activation of beige adipose tissue through the activation of the HSF1-A2B1 transcriptional axis [59]. Heat shock factor 1 (HSF1) plays a regulatory role in modulating the levels of PGC-1α both transcriptionally and post-transcriptionally in response to obesity and aging, contributing to the maintenance of cellular homeostasis. Additionally, non-lethal hyperthermia-induced perturbations upregulate HSF1 and result in mitohormesis, yielding beneficial outcomes in the context of aging [60,61,62,63] (Figure 3).

### 3.3. Regular Exercise

Epidemiological studies unequivocally demonstrate that physical inactivity is a significant contributor to abdominal adiposity. Nevertheless, regular exercise has long been recognized as a therapeutic approach for managing obesity and diabetes, leading to a reduction in abdominal adiposity and mitigating metabolic syndrome. Serving as a valuable strategy in primary care and community health, regular exercise proves beneficial in addressing aging and age-related diseases. The enduring enhancement in glucose clearance induced by long-term exercise training persists for a considerable duration. 

In summary, regular exercise (e.g., resistance training, moderate-intensity endurance training) plays a crucial role in counteracting the development of obesity and diabetes stimulated by aging [64]. The research indicates that engaging in physical activity can lead to a reduction in food intake, low-grade inflammation, and lipogenesis, thereby alleviating insulin resistance in response to both obesity and aging [65]. In elderly individuals (aged 65 years and over) who engage in prolonged endurance exercise (15 consecutive days of biking for 7–9 h/day at 63% and 65% of maximal heart rate), there was an increase in macrophage content and mitochondrial respiration in adipose tissue [66]. A 12-month exercise program revealed that prolonged exercise training (a combination of aerobic and strength training for a minimum of 150 min per week) may signify a certain degree of remodeling in adipose tissue among older patients (age 41–81 years) with coronary artery disease and diabetes [67]. Furthermore, both endurance (high-intensive interval exercise performed on an ergometer bicycle, 3 times a week for 45 min) and resistance exercise (a 45-min interval-type, medium-load, high-repetition, time-based training, 3 times a week) decrease the mass of epicardial adipose tissue in individuals with abdominal obesity but also mitigate obesity-induced cardiac fat accumulation [68]. Nevertheless, the precise mechanism by which exercise ameliorates metabolic disorders induced by aging and obesity remains not fully identified. 

## 4. The Potential Role of Regular Exercise in Aged Adipose Tissue

**Figure 4:**
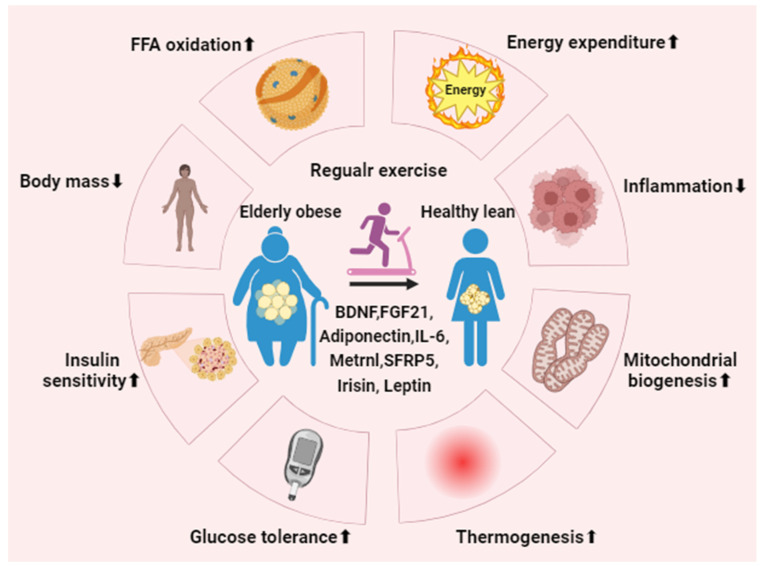
Regular exercise mitigates metabolic syndrome. Consistent physical activity promotes the activation and generation of beige adipocytes in individuals over the age of 65 with obesity by regulating associated exerkines and adipokines. Ultimately, this process enhances metabolic performance, leading to notable benefits such as decreased inflammation and body mass, heightened energy expenditure, improved glucose tolerance, increased insulin sensitivity, and the stimulation of mitochondrial biogenesis and UCP1-dependent thermogenesis. BDNF, brain-derived neurotrophic factor; FGF21, fibroblast growth factor 21; Metrnl, meteorin-like protein; SFRP5, secreted-frizzled-related protein 5. Up arrow signifies an increase, while down arrow indicates a decrease.

Regular exercise, such as resistance ladder-climbing exercise, running-wheel exercise at a constant speed of 18 m/min for 60 min daily, 5 d/wk for 8 wk, has been shown to induce significant alterations in the morphology and function of AT, particularly in response to metabolic diseases. These changes include an increase in fat browning, a reduction in adipocyte hypertrophy, and improvements in glucose and lipid metabolism in AT [69,70,71,72]. Moreover, 2-week running-wheel exercise training not only triggers a phenotypic transformation of AT, shifting it from primarily storing energy as white adipocytes to thermogenic beige adipocytes, especially in the context of obesity and diabetes. Additionally, it enhances processes such as FFA oxidation, insulin sensitivity, alleviation of oxidative stress, as well as the promotion of mitochondrial biogenesis and function [10,73] (Figure 4). 

### 4.1. White Adipose Tissue

WAT, being a highly prevalent form of AT, is distributed throughout nearly every region of the body [74]. Nevertheless, the functional decline of AT in the context of obesity and diabetes is involved in a reduction in AT plasticity. This is evident in the significant decrease in AT metabolism and alterations in phenotype to meet the demands of the organism [14]. The maladaptive remodeling of AT, marked by heightened fibrosis proliferation and a pro-inflammatory response, is triggered by a breakdown in angiogenesis and local hypoxia [75,76]. As a result, adipose tissue becomes insulin-resistant, inflamed, fibrotic, and dysfunctional, particularly in the context of aging.

Numerous studies have demonstrated that exercise (e.g., high-intensity interval treadmill training 3 times per week for 60 min each session over 8 months, or walking combined with arm aerobics 3 times per week for 4 months) has a profound impact on systemic metabolism by adapting to various tissues, including the heart [77,78,79], liver [80], skeletal muscle [81,82], and AT [7,64,83,84,85,86]. AT depots, which play crucial roles in metabolism, are implicated in mitochondrial biogenesis, glucose metabolism, and FFA oxidation and uptake in response to exercise. These depots include inguinal WAT, perigonadal WAT, and interscapular BAT [5]. Routine aerobic exercise (e.g., 6–8 weeks of swimming or treadmill training at 75–85% of V_O2,max_ for 60 min per day, 5 days per week) brings about a significant reduction in WAT and a substantial increase in BAT in both mice and humans. This effect is achieved through the stimulation of various growth factors and cytokines, fostering the proliferation and differentiation of brown preadipocytes [87,88]. In WAT, regular exercise (e.g., swimming exercise training for 90 min daily, 5 days/week for 4 weeks, or resistance training 3 times a week for 60 min each session over 6 weeks) leads to a considerable decrease in adipocyte size [89], an increase in mitochondrial biogenesis [90,91,92], regulation of adipokine secretion [93,94], and an overall enhancement of whole-body metabolic health [95]. Furthermore, 12-month treadmill exercise training (starting at 3 m/min for 5 min, increasing to 4.8 m/min for 5 min, and reaching a maximum of 7.2 m/min for 20 min, with a 0% slope) induces adaptability in WAT, as indicated by elevated FFA oxidation and a reduction in the impact of inflammation, achieved through the regulation of pro/anti-inflammatory gene expression and the infiltration of macrophages [96]. Furthermore, exercise training contributes to the improvement of mitochondrial biogenesis and thermogenesis by facilitating the transformation of white adipocytes into beige adipocytes in WAT, counteracting the effects of aging and obesity [96]. 

### 4.2. Brown Adipose Tissue

BAT, a specialized heat-generating organ rich in mitochondria, is crucial for maintaining body temperature in cold conditions [97]. Mitochondrial biogenesis and function in BAT play a pivotal role in thermoregulation and metabolic processes. Regular exercise has been shown to enhance UCP1 content, mitochondrial respiration and activity, and upregulate genes associated with mitochondrial biogenesis in BAT [5]. Consistent physical activity (voluntary wheel-running training for 4 weeks) significantly reduces fat mass and body weight gain, enhances energy expenditure, and elevates UCP1 expression in BAT by activating the AMP-activated protein kinase (AMPK) signaling pathway [98]. UCP1, responsible for dissipating the proton motive force as heat, augments the energy metabolism of mitochondria in BAT, contributing to adaptive non-shivering thermogenesis (NST) [97]. The presence and function of BAT are reported to be diminished by metabolic diseases [99] and aging [100,101,102,103]. Nonetheless, functional BAT has been shown to reduce oxidative stress, alleviate pathological cardiac hypertrophy, and enhance cardiac function by promoting the release of exerkines such as FGF-21 and IL-6 [104,105]. 

Endurance exercise training or physical activity in young sedentary adults enhances BAT volume, playing a significant role in regulating glucose metabolism in an intensity-dependent manner. This study demonstrates that the BAT response becomes stronger with increasing exercise intensity [8]. Furthermore, voluntary wheel-running training induces alterations in lipid metabolism in AT by modifying the lipidomes of both WAT and BAT. This is evident in the reduction of specific molecular species of phosphatidic acid (PA), phosphatidylcholines (PC), phosphatidylethanolamines (PE), and phosphatidylserines (PS) in WAT, and the increase in specific molecular species of PC and PE in BAT. There is also a decrease in the majority of triacylglycerols (TAGs) in both WAT and BAT [4]. Additionally, physical activity or exercise training (e.g., endurance exercise, resistance exercise) enhances mitochondrial activity, glucose uptake, insulin sensitivity, and thermogenesis in BAT [7,72,84,106,107,108,109]. Cardiolipin (CL), a mitochondrial phospholipid, is essential for mitochondrial metabolism and structural integrity [110,111,112,113,114]. Moreover, CL serves as a key effector in the thermogenic programs of brown and beige adipocytes and is involved in insulin sensitivity in AT [115]. Conversely, the depletion of CL in brown and beige adipocytes impairs thermogenesis and glucose metabolism, resulting in reduced insulin sensitivity [115].

### 4.3. Beige Adipose Tissue

In addition to BAT, cells within WAT undergo adaptive thermogenesis in response to cold exposure or prolonged exercise training and are referred to as beige adipocytes. The development of beige adipocytes is regulated by factors such as PR domain containing 16 (PRDM16), peroxisome proliferator-activated receptor gamma (PPARγ), and CCAAT-enhancer-binding proteins (C/EBP) [116]. Beige cells represent an inducible profile of thermogenic adipocytes that can be activated by various stimuli, enhancing their capacity for fuel oxidation and thermogenesis. These stimuli include exercise, cold exposure, local hyperthermia therapy, and β-adrenergic intervention [59,116,117,118]. Research has shown that sustained physical activity and exercise induce the beiging of WAT by modulating the secretion of brain-derived neurotrophic factor (BDNF), irisin, PGC-1α, interleukin-6 (IL-6), and meteorin-like protein (Metrnl) [95,119,120]. Moreover, exercise activates signaling pathways associated with beiging in WAT, including the Wnt/β-catenin signaling pathway—a novel pathway crucial for driving the adipocyte population required for beiging. Additionally, exercise influences PGC-1α-related pathways, which mediate mitochondrial biogenesis and function [121].

Regrettably, aging results in a reduction in the mass of BAT in adult humans [122,123,124], and it diminishes cold and exercise-induced beiging in aged mice. This is evidenced by a decrease in the expression of transcriptional markers associated with beige adipocytes [50,116,125,126]. The number of senescent cells increases while the differentiation of beige adipocytes decreases in aged mice and middle-aged humans. This is indicated by elevated transcriptional factors of senescence in WAT, including p16^Ink4a^, p21, and insulin-like growth factor binding protein 5 (IGFBP5). Furthermore, this phenomenon leads to an increase in glucose content and mitophagy, coupled with an incapacity to regulate the adaptation of body temperature in response to cold exposure. These findings demonstrate that cellular senescence plays a pivotal role in the age-induced decline of beige adipocyte generation [50,127,128,129]. The study revealed that sustained stimulation of β-adrenergic agonists induces beiging in middle-aged mice [16,130]. Various factors act as transcriptional regulators influencing differentiation in adipose tissues in response to aging.

## 5. Effect of Exercise-Induced Adipokine in Aged Adipose Tissue

Aging induces structural, compositional, and functional changes in AT, characterized by reduced adipogenesis, alterations in the immune cell profile, and increased inflammation [131]. As the largest endocrine gland, AT releases various cytokines that regulate metabolic responses, encompassing pre-production, adipogenesis, glucose and lipid homeostasis, inflammation, and several other physiological functions [132]. Aging exerts a negative regulatory impact on the secretion of adipokines, as evidenced by an increase in proinflammatory adipokines (e.g., leptin, resistin, chemerin, retinol-binding protein 4, lipocalin 2, CCL2, IL-1β, IL-6, IL-12, IL-18, and TNF-α) [133,134,135,136,137,138], coupled with a decline in anti-inflammatory mediators (e.g., adiponectin, vaspin, secreted-frizzled-related protein 5, omentin-1, and C1q/TNF-related proteins) [139,140,141,142] (Table 1). Nevertheless, regular exercise can enhance the secretion of adipokines and mitigate the morphology and function of AT in response to metabolic diseases. This includes promoting fat browning, reducing adipocyte hypertrophy, improving FFA oxidation, insulin resistance, and enhancing mitochondrial homeostasis in aging AT [69,70,71,72].

Adipokines such as adiponectin and spexin, which decrease with aging in AT, play a crucial role in insulin resistance and are associated with the onset of diabetes and other metabolic disorders [64,143,144]. Aging adipose tissue impacts the secretion of adipokines, promoting a chronic state of low-grade systemic inflammation [139]. The exerkine IL-6, when exposed to acute inflammatory stress, is significantly increased with aging in AT. The age-dependent secretion of IL-6 is regulated by the autocrine/paracrine action of IL-1β in aged AT [145]. BAT, fulfilling endocrine functions, also releases hormones known as batokines, which play a role in regulating energy balance, glucose uptake, lipid metabolism, and thermogenesis [146,147,148]. Batokines are exercise-related humoral factors originating from BAT, exerting local autocrine or paracrine effects. These factors include peptides, metabolites, lipids, or microRNAs [11]. Multiple studies demonstrate that exercise training or physical activity induces the differentiation of white adipocytes into functionally equivalent brown adipocytes, enhancing BAT function. Additionally, brown adipose tissue plays a role in mediating exercise performance [95,146,147]. Nevertheless, treadmill exercise training enhances energy metabolism in response to cold exposure, as demonstrated by the promotion of mitochondrial biogenesis, reduction in oxidative stress, and increased exercise capacity [84]. Moreover, the study reveals that small extracellular vesicles secreted from BAT not only promote metabolism within BAT but also regulate cardiomyocyte survival and participate in the response to exercise and myocardial ischemia/reperfusion injury. This is evidenced by the suppression of the proapoptotic MAPK pathway [7].

**Table 1:** The impact of adipokine in aged AT.

Adipokines	Main Mechanism	Main Biological Action	Target	Refs
Leptin	Srebp-1c/FGF21/ PGC-1α	Regulates FA biosynthesis and mitochondrial biogenesis	AT	Kobayashi, M., et al. [149]
Resistin	CRP/IL-6/TNF-α	Associates with aging-related cardiovascular disease	Heart	Gencer, B., et al. [135]
Chemerin	PRDM16/CPT1/ DIO2	Regulates formation and function of BAT	BAT	Zhang, Y., et al. [136]
RBP4	JNK/TNF/IL-1β	Causes insulin resistance and inflammation by activating innate immunity	AT	Moraes-Vieira, P. M., et al. [137]
LCN2	mTORC1/ERK	Regulates mitochondrial bioenergetics	BAT	Su, H., et al. [138]
IL-6	IL-1β/TNF-α	Impact age-associated inflammatory diseases	AT	Starr, M. E., et al. [145]
Adiponectin	ARG1/TNF	Mediates the anti-inflammatory effects of niacin	AT	Graff, E. C., et al. [150]
Vaspin	ANGPTL4/DNA methylation	Reduces inflammation and activists BAT	BAT	Weiner, J., et al. [140]
SFRP5	JNK/Wnt	Regulates inflammation and obesity-related complication	AT	Koutaki, D., et al. [142]
CTRPs	AMPK/Akt, ERK	Mitigates heart failure by improving inflammation	Heart	Shanaki, M., et al. [151]
Omentin-1	AMPK/Akt	Improves cardiovascular disease by mitigating inflammation	Heart	Xu, F., et al. [152]

CRP, C-reactive protein; RBP4, retinol binding protein 4; LCN2, lipocalin 2; SFRP5, secreted-frizzled-related protein 5; CTRPs, C1q/TNF-Related Proteins; ARG1, arginase 1; CPT1, carnitine palmitoyltransferase 1.

## 6. Conclusions

Aging of adipose tissue is linked to alterations in structure, composition, and function, encompassing changes in adipokine secretion, reduced adipogenesis, shifts in immune cell profile, heightened cellular senescence, increased insulin resistance, elevated inflammation, and enhanced fibrosis. As the largest endocrine gland, adipose tissue releases a variety of cytokines that regulate metabolic responses. Adipokines released through regular exercise play potential roles in mitigating metabolic diseases, improving glucose and lipid metabolism, reducing inflammation and fibrosis, and promoting fat browning and thermogenesis in adipose tissue. Here, we provide a comprehensive review of the potential effects of regular exercise on aged adipose tissue by regulating associated exerkines and adipokines. Ultimately, this process enhances metabolic performance, leading to notable benefits such as decreased inflammation and body mass, heightened energy expenditure, improved glucose tolerance, increased insulin sensitivity, and the stimulation of mitochondrial biogenesis and thermogenesis. Thus, the potential exists for designing therapeutic interventions based on these molecular mechanisms.

## Data Availability

Not applicable.
